# Le Docteur Noir

**Published:** 1859-06

**Authors:** 


*Le Docteur Noir.*—In one of our late numbers, we mentioned the case of the black doctor who has been making so much noise with his cancer curing at Paris, and has even as wide-spread a notoriety as the celebrated Dr. Fell, of Middlesex Hospital fame. So distinguished had this quack become. that a portrait and biographical sketch of him appeared some time ago in one of our illustrated weeklies. From this we see that he is the son of a rich native of Java; that he went to Holland to study medicine, where he was a "student of remarkable promise." After his return to his own country, he discovered or purchased the secret of an infallible remedy for cancer, with which he went to Paris, and has been performing the most miraculous cures. One of his cases is detailed in the "*Courrier des Etats Unis*," and is so good that we cannot forbear publishing a full translation of it, which we made at the time it appeared. The patient was the musician Sax, the inventor of the Sax horns.

"*The Black Doctor.*—Paris is now very much occupied with a mysterious physician, M. Vries, called "the black Doctor of Java," who accomplishes, they say, miraculous cures. This extraordinary man has become the fashion, and his consultations are paid for at enormous prices, to the great indignation of the doctors of the Faculty, who have entered an action against their Indian rival for the illegal practice of medicine. A petition has been addressed to the Emperor by many of the most influential members of the section of music of the *Institut*, and by very distinguished persons cured by the black doctor, in order to stop or arrest the processes.

Among the persons known, which this doctor of the "thousand and one nights" has cured, is cited M. Adolphe Sax, who was saved from a horrible and incurable malady. Adolphe Sax had a melanotic or black cancer of the lip with which he had suffered the agonies of the damned for many years, (since 1853), MM. Ricord and Velpeau having given up all hope of curing Sax, his friends believing him lost.

Black cancer, fortunately quite rare, says the "*Messages de Paris*," leads invariably to death those whom it attacks, whether it be treated by diet and internal remedies, or whether it be submitted to a frightful surgical operation. Sax had arrived at that point where it was necessary to make him undergo that operation, to remove the lips and cheeks and disfigure him forever, without hope of saving him. It was then that Sax went to consult the black doctor. To have an idea of the state in which Sax was at that time, it is necessary to read the horrible details, the learned and terrific description given of his disease by Dr. Déclat, in the "*Moniteur des Hôpitaux*."

The black doctor examined Sax, and told him "You have black cancer . . . but me to cure you" (*vous avoir cancere noire . . . Mais moi guérir vous*). Sax was lost, and gave himself entirely into the hands of the black doctor. His treatment commenced in the month of June last. The diet, a treatment of severe regimen; in short, internal remedies cured Sax in less than six months.

Nov. 14, Sax's complaint was continually worse, but the confidence of Dr. Vries was unabated. On that day the Doctor said to Sax, "You to have made portrait in Photograph" (*vous faire faire portrait à Photographie*). Sax neglected this recommendation, which he thought singular. The next day the doctor said, "If to-morrow you no have to photograph you, me come no more, and leave you" (*Si demain vous pas fait photographier vous, moi plus venir et lassier vous*). Sax had his portrait taken and gave it to Dr. Vries, who said to him, "I wanted a portrait of you thus, for when you cure, others no think how you be to-day; in a few days you cure" (*J'ai voulu portrait à vous ainsi,*

*parce que quand vous guéri, autre pas croire comment être aujourd'hui, etc*). In fine, a few days after, the inflammation became horrible; the tumor frightful; one would have said that a burning fire devoured the face of the unfortunate Sax. But extraordinary to say, the fire only ate up the disease; the cancer alone burned in its substance, and its *debris* fell carbonized in fragments, of which some were the size of a cherry. To-day there is not the least scar of the horrible disease before which all the *savants* recoiled.

In recognition of this miraculous cure, the friends of Sax offered the black doctor a banquet, at which M. Mathiêu presided. The dinner was of one hundred and fifty covers, and was arranged by M. M. Sax and Berlois. Among the guests figured M. M. Ambroise Thomas, Halévy, Auber, as well as other musical celebrities. *La musique des guides*, composed by Auber expressly for the occasion, was performed during the repast."

This remarkable imposter had such success and notoriety that M. Velpeau was induced to give up to his charge a certain number of cases of undoubted cancer at *La Charité*, the results of which are such as might be expected. Why such quacks impose upon the public, we are at a loss to say, but the actual test is always the best, and the only argument, in the eyes of the world, which we can have against them. The following is the result of this trial by M. Velpeau, which we have cut from one of our exchanges. We have published this long account of the case of Sax, hoping that it will be as amusing to our readers as it was to ourselves.

"The attention of the public has been lately directed to the relation of some wonderful cures said to have been performed in some cases of cancer, by a medical man of color named Vries. The cures at length became so much talked of, that M. Velpeau, surgeon at the hospital of La Charité, member of the Institute of the Academy of Medicine, was desirous of ascertaining the correctness of the accounts given, and of arriving at a correct estimate of the real value of the specific used by M. Vries. For this purpose an offer was made him to take under his exclusive treatment sixteen cases of confirmed cancer, at La Charité, which he accepted, and those patients have been for two months subjected to the use of what he calls his antidote against that dreadful disease. This treatment commenced on the 27th January, and on the 29th March, M. Velpeau read to the Academy of Medicine a report of the result. After stating that every arrangement had been made, and the most positive directions given, that no one belonging to the hospital should in any way interfere with the patients placed under the care of M. Vries, and that all the orders he might give should be punctually attended to, M. Velpeau states that nothing has been effected to bear out the pretensions of M. Vries; that none of the patients have been cured; that one of them, a female, died at the end of ten days, and that with all the others the disease has followed its ordinary course, and after two months' treatment the patients have not shown any improvement. M. Velpeau sums up his report by the following conclusions: That no antidote for cancer has yet been discovered; that M. Vries has effected no cure on any of the patients entrusted to him in the hospital; and that he has never cured and never can cure a case of cancer anywhere. The Academy unanimously decided that the report of M. Velpeau should be sent to the Minister of Justice, and that M. Vries should be no longer admitted to the hospital."

